# Chiari I malformation risk across anatomical subtypes of sagittal synostosis

**DOI:** 10.3389/fsurg.2026.1949189

**Published:** 2026-10-23

**Authors:** Chenxu Kong, Linzhao Min, Jiajia Wang, Jia Wei, Zhenjiang Li, Xiaoqiang Wang

**Affiliations:** 1 Huaihe Hospital, Henan University, Kaifeng, China; 2 Xinhua Hospital, School of Medicine, Shanghai Jiao Tong University, Shanghai, China

**Keywords:** chiari type i malformation, posterior fossa volume, quantitative imaging, sagittal synostosis, tentorial angle

## Abstract

**Background:**

Sagittal synostosis is increasingly recognized as a heterogeneous condition. Whether distinct anatomical phenotypes differ in Chiari type I malformation (CM-I) burden, and how posterior fossa morphology contributes to this heterogeneity, remains unclear.

**Methods:**

We retrospectively studied 241 children with sagittal synostosis who underwent preoperative three-dimensional CT volumetry and MRI between January 2023 and December 2025. Patients were classified as anterior fossa–involved (Group A, *n* = 63), posterior fossa–involved (Group B, *n* = 53), or isolated sagittal synostosis (Group C, *n* = 125). Residualized posterior fossa volume (PFV_adj) was derived from PFV regressed on preoperative intracranial volume and age at surgery. ANCOVA and sequential logistic regression models were used, with Firth penalized regression as a sensitivity analysis.

**Results:**

CM-I prevalence differed across subtypes (*P* = 0.012) and was highest in Group B (28.3%) versus Group A (11.1%) and Group C (12.0%). In the subtype-only model, Group B had higher CM-I odds than Group C (OR = 2.89, 95% CI 1.29–6.48; *P* = 0.010). Group B also showed significantly lower PFV_adj (*P* < 0.001). In morphology-adjusted analyses, greater tentorial angle (OR = 3.20 per 5°, 95% CI 2.10–4.88; *P* < 0.001) and lower PFV_adj (OR = 0.74 per 10 mL, 95% CI 0.62–0.89; *P* = 0.002) were associated with CM-I. Model AUC increased numerically from 0.869 to 0.885 after adding PFV_adj. Firth regression yielded consistent results.

**Conclusions:**

The posterior fossa–involved phenotype carries the greatest overall CM-I burden and is characterized by relative posterior fossa deficiency and a steeper tentorial configuration. PFV_adj provides a developmentally adjusted measure that complements anatomical subtype classification.

## 1 Introduction

Craniosynostosis is a congenital craniofacial anomaly characterized by premature fusion of one or more cranial sutures, with an estimated incidence of approximately 1 in 2,500 live births (1). Sagittal synostosis is the most common subtype, accounting for ∼50% of all cases (2). It typically presents as early fusion of the sagittal suture, leading to the characteristic scaphocephalic cranial deformity, defined by an increased anteroposterior cranial dimension with restricted lateral expansion of the parietal and temporal regions (3). In some patients, synostosis may be confined to the anterior or posterior segment of the sagittal suture rather than involving the entire suture. Beyond cosmetic considerations, approximately 15%–40% of patients may develop elevated intracranial pressure (ICP), which, if not recognized and treated in a timely manner, can contribute to long-term neurocognitive impairment (4, 5). Accordingly, the primary goal of surgical management is to prevent complications related to raised ICP (6).

Chiari type I malformation (CM-I) is defined as downward herniation of the cerebellar tonsils through the foramen magnum into the spinal canal, most commonly diagnosed on imaging when tonsillar descent is ≥5 mm (7). Its pathophysiology is frequently linked to a relatively small osseous posterior fossa and consequent crowding, with caudal displacement of posterior fossa contents due to reduced available space (8, 9). Prior studies have shown a high prevalence of Chiari malformation among patients with complex or syndromic craniosynostosis, and certain nonsyndromic single-suture subtypes—particularly lambdoid synostosis—also demonstrate a strong association with CM-I (10). In contrast, isolated sagittal synostosis has historically been considered less commonly complicated by CM-I; however, recent large cohort data indicate that CM-I is not rare in this population. For example, among 377 patients with isolated sagittal synostosis, the overall prevalence of CM-I was approximately 5.8%, with a markedly higher rate in those presenting later (9.2%) compared with those presenting earlier (1.0%) (11). Collectively, these findings suggest that a non-negligible subset of patients with sagittal synostosis develop CM-I, potentially influenced by suture phenotype and timing of presentation. Given that distinct sagittal synostosis subtypes may differentially shape cranial morphology and posterior fossa volume, clarifying their relationship with CM-I has important clinical implications.

Neuroimaging constitutes the cornerstone for accurate phenotypic classification of craniosynostosis and for risk stratification of CM-I. Three-dimensional computed tomography (3D CT) has long been regarded as the reference standard for assessing suture fusion and cranial morphology, providing high-resolution visualization of osseous structures and enabling multiplanar and volumetric reconstructions. These capabilities facilitate quantitative characterization of cranial geometry, measurement of intracranial volume (ICV), and derivation of posterior fossa–related volumetric metrics, thereby supporting preoperative planning (12).n parallel, given children's heightened sensitivity to ionizing radiation, low-dose protocols and adherence to the “as low as reasonably achievable” (ALARA) principle have been increasingly emphasized (13). Complementing CT, magnetic resonance imaging (MRI) offers excellent soft-tissue and cerebrospinal fluid contrast without ionizing radiation and is therefore central to the evaluation of CM-I and associated posterior fossa/spinal abnormalities, including tonsillar herniation and syringomyelia. In patients with neurological symptoms, clinical or radiological indicators of elevated intracranial pressure, or complex phenotypes, a combined CT–MRI approach is generally considered warranted to achieve a comprehensive assessment (14).

Accordingly, this study will leverage preoperative imaging to develop a quantitative, anatomy-based phenotyping framework that captures the clinical heterogeneity of sagittal synostosis. Using standardized measurements derived from 3D CT reconstructions, we will quantify intracranial volume (ICV), posterior fossa volume (PFV), and key geometric parameters (e.g., tentorial angle). These metrics will be integrated with MRI-based determination of CM-I to compare CM-I risk across three sagittal synostosis subtypes: sagittal synostosis with anterior involvement (Group A), sagittal synostosis with posterior fossa restriction (Group B), and isolated sagittal synostosis (Group C). We will further develop a multivariable predictive model to support preoperative risk stratification and inform individualized imaging surveillance strategies.

## 2 Methods

### 2.1 Study design and participants

We conducted a single-center retrospective observational cohort study of children with sagittal craniosynostosis who underwent surgical treatment at the Department of Pediatric Neurosurgery, Xinhua Hospital, Shanghai Jiao Tong University School of Medicine, between January 2023 and December 2025. Patients were not selected according to CM-I status; rather, eligible patients were identified from the defined surgical cohort during the prespecified study period and subsequently categorized according to anatomical phenotype. To enhance cohort homogeneity and minimize confounding, we applied the following exclusion criteria: (1) syndromic craniosynostosis (e.g., Apert, Crouzon, or Pfeiffer syndromes), given their distinct cranial base developmental abnormalities and potentially different mechanisms underlying cerebellar tonsillar descent (Chiari malformation); (2) complex multi-suture synostosis not meeting the prespecified phenotypic definitions (e.g., sagittal synostosis combined with bilateral coronal synostosis); (3) incomplete clinical or imaging data, particularly absence of preoperative brain MRI for CM-I assessment or missing key imaging-derived measurements; and (4) history of prior cranial surgery performed at an outside institution, to avoid bias introduced by previous interventions on intracranial volume or cranial base anatomy. A total of 241 eligible patients were included in the final analysis. This retrospective study was approved by the Ethics Committee of Xinhua Hospital, Shanghai Jiao Tong University School of Medicine [Approval No. (XHEC-C-2026-101-2)].

### 2.2 Clinical data collection and imaging measurements

Baseline clinical characteristics were extracted from medical records and the picture archiving and communication system (PACS), including sex, gestational age at birth (to ascertain prematurity), birth weight, and age at surgery (months). All participants underwent preoperative thin-slice CT and MRI. Imaging-derived variables included: Volumetric metrics: intracranial volume (ICV) and posterior fossa volume (PFV) were quantified based on 3D CT reconstruction. The PFV-to-ICV ratio (PFV/ICV) was additionally calculated to index the relative degree of posterior fossa restriction. Cranial base morphology: tentorial angle and basal angle were measured as standardized geometric parameters. Ventricular assessment: the Evans index was computed to quantify lateral ventricular enlargement; hydrocephalus was defined radiographically as an Evans index > 0.30. In addition, the presence of other neuraxial abnormalities (e.g., syringomyelia) was recorded. To ensure measurement reliability, all key imaging variables were initially measured on the original imaging datasets by one investigator, followed by independent double data entry and cross-validation by two investigators. Discrepant qualitative imaging features were adjudicated with input from a neuroradiology specialist.

### 2.3 Phenotypic classification of sagittal synostosis

Based on preoperative imaging features and intraoperative assessment of suture involvement and cranial fossa compensatory patterns, patients were categorized into three anatomical subtypes: Type A: sagittal synostosis with anterior fossa involvement (*n* = 63). This subtype was defined by sagittal synostosis accompanied by prominent anterior cranial base dysmorphology, characterized by bilateral frontal bossing and/or excessive frontal expansion; metopic suture fusion could be present in a subset of cases. Type B: sagittal synostosis with posterior fossa involvement (*n* = 53). This subtype was defined by sagittal synostosis with substantial involvement of posterior cranial base structures, resulting in restricted compensatory capacity of the posterior fossa; imaging could demonstrate occipital compression and/or abnormal tentorial position, and some cases showed unilateral or bilateral lambdoid suture fusion. Type C: isolated sagittal synostosis (n = 125). This reference group was defined by isolated, complete premature fusion of the sagittal suture without involvement of the metopic, coronal, or lambdoid sutures. Patients typically exhibited classic scaphocephaly without overt evidence of cranial fossa developmental imbalance.

### 2.4 Primary outcome

The primary outcome was the presence of Chiari type I malformation (CM-I) on preoperative imaging. CM-I status was coded as a binary variable (CM-I present vs. absent) and used as the dependent variable in multivariable modeling.

Standardization of Posterior Fossa Volume: Residualized PFV (PFV_adj).

Because posterior fossa volume during infancy and childhood is strongly influenced by overall cranial size and age-related growth, we derived a residualized measure of posterior fossa volume (PFV_adj). A linear regression model was fitted with PFV as the dependent variable and ICV_pre and age at surgery as predictors:

PFV=α+β1⋅ICVpre+β2⋅Age+ε.

The residual term (*ε*) from this model was defined as PFV_adj (mL). PFV_adj therefore represents the deviation of the observed PFV from its expected value at a given ICV_pre and age. Negative values indicate relative posterior fossa volume deficiency, whereas positive values indicate relative volume excess.

### 2.5 Statistical analysis

All statistical tests were two-sided, with *P* < 0.05 considered statistically significant. Continuous variables were summarized as median [P25, P75] according to their distributions and compared across the three subtypes using the Kruskal–Wallis test. Categorical variables were summarized as *n* (%) and compared using the *χ*^2^ test or Fisher's exact test, as appropriate.

To account for age-related cranial growth and overall cranial size, PFV_adj was derived using the residualization approach described above. Differences in PFV among anatomical subtypes were additionally evaluated using analysis of covariance (ANCOVA), with PFV as the dependent variable, subtype as the main grouping factor, and ICV_pre and age at surgery as covariates. Covariate-adjusted least-squares means (LS-means) were estimated, and subtype-specific fitted relationships were illustrated with ICV_pre and age fixed at the cohort mean.

Associations with CM-I were evaluated using three sequential logistic regression models. Model 1 included anatomical subtype only and was used to characterize the overall subtype–CM-I association without conditioning on posterior fossa morphometric variables. Model 2 included subtype, tentorial angle, Evans index, age at surgery, and hydrocephalus. Model 3 additionally included PFV_adj.

For clinical interpretability, effect sizes were scaled per 5° increase in tentorial angle, per 0.05 increase in Evans index, per 12-month increase in age at surgery, and per 10-mL increase in PFV_adj. Results are presented as odds ratios (ORs) with 95% confidence intervals (CIs).

A total of 37 CM-I events were observed. The numbers of events per non-intercept parameter were 18.5, 6.2, and 5.3 for Models 1, 2, and 3, respectively. Given the relatively low EPV in Models 2 and 3, Firth bias-reduced penalized logistic regression was additionally performed as a sensitivity analysis to assess the robustness of the estimates to small-sample and sparse-data bias.

Model discrimination was assessed using the area under the receiver-operating-characteristic curve (AUC). Because tentorial angle and PFV_adj quantify morphologic features closely related to the posterior fossa–involved subtype B phenotype, subtype coefficients in Models 2 and 3 were interpreted as conditional associations rather than estimates of the overall subtype effect.

## 3 Results

### 3.1 Baseline characteristics and phenotypic distribution

A total of 241 children were included, comprising 63 in Group A, 53 in Group B, and 125 in Group C. Gestational age at birth differed significantly across groups (*P* = 0.026), whereas birth weight (*P* = 0.291) and age at surgery (*P* = 0.578) were comparable. Among unadjusted preoperative imaging metrics, preoperative intracranial volume (ICV_pre) did not differ significantly (*P* = 0.160), and posterior fossa volume (PFV) showed only a borderline between-group trend (*P* = 0.075). In contrast, morphological parameters demonstrated marked heterogeneity: both the tentorial angle and the PFV/ICV ratio differed substantially across groups (both *P* < 0.001), while the basal angle and Evans index were relatively similar. Notably, after deriving residualized PFV (PFV_adj) using the covariate-adjusted residual approach, between-group differences became more pronounced (*P* < 0.001). Group B exhibited a clear negative deviation in PFV_adj, indicating relative posterior fossa underdevelopment after accounting for age and overall cranial size, whereas PFV_adj values in Groups A and C tended to be closer to or above the expected range (Table 1).

**Table 1:** Baseline characteristics of patients with sagittal synostosis by anatomical subtype (A/B/C).

Variable	A	B	C	*P* value
Gestational age at birth (days)	268.00 [264.00, 276.00]	266.00 [263.00, 273.00]	273.00 [265.00, 276.00]	0.026
Birth weight (g)	3,443.00 [3,401.50, 3,466.00]	3,454.00 [3,434.00, 3,472.00]	3,451.00 [3,424.00,3,467.00]	0.291
Age at surgery (months)	31.33 [18.88, 43.33]	24.77 [15.33, 41.27]	28.50 [14.83, 40.90]	0.578
Preoperative intracranial volume, ICV (mL)	1,347.73 [1,161.51, 1,475.49]	1,356.95 [1,242.92, 1,495.22]	1,300.12 [1,125.21,1,463.62]	0.160
Posterior fossa volume, PFV (mL)	183.96 [160.02, 195.23]	166.23 [149.93, 181.83]	175.34 [152.02, 193.65]	0.075
PFV_adj (mL; adjusted for ICV and age)	6.65 [−6.54, 13.49]	−11.53 [−18.65, −4.53]	5.28 [−4.61, 17.40]	<0.001
PFV/ICV ratio	0.14 [0.12, 0.14]	0.12 [0.12, 0.12]	0.14 [0.12, 0.15]	<0.001
Tentorial angle (°)	35.00 [31.00, 39.00]	38.00 [32.00, 50.00]	35.00[30.00, 36.00]	<0.001
Basal angle (°)	134.00 [126.50, 137.50]	136.00 [130.00, 139.00]	134.00 [128.00, 137.00]	0.190
Evans index	0.26 [0.24, 0.27]	0.26 [0.25, 0.27]	0.27 [0.25, 0.28]	0.163
Female sex	15 (23.8%)	9 (17.0%)	29 (23.2%)	0.606
Preterm birth	4 (6.3%)	6 (11.3%)	4 (3.2%)	0.104
Hydrocephalus	3 (4.8%)	2 (3.8%)	2 (1.6%)	0.435
CM-I present	7 (11.1%)	15 (28.3%)	15 (12.0%)	0.012

Values are median [P25, P75] or *n* (%), as appropriate. PFV_adj denotes residualized PFV after adjustment for preoperative ICV and age at surgery.

### 3.2 Between-group differences in the prevalence of Chiari type I malformation

The prevalence of Chiari type I malformation (CM-I) differed significantly across the three subtypes (*P* = 0.012). Group B exhibited the highest CM-I rate at 28.3% (15/53), compared with 11.1% (7/63) in Group A and 12.0% (15/125) in Group C (Figure 1).

**Figure 1:**
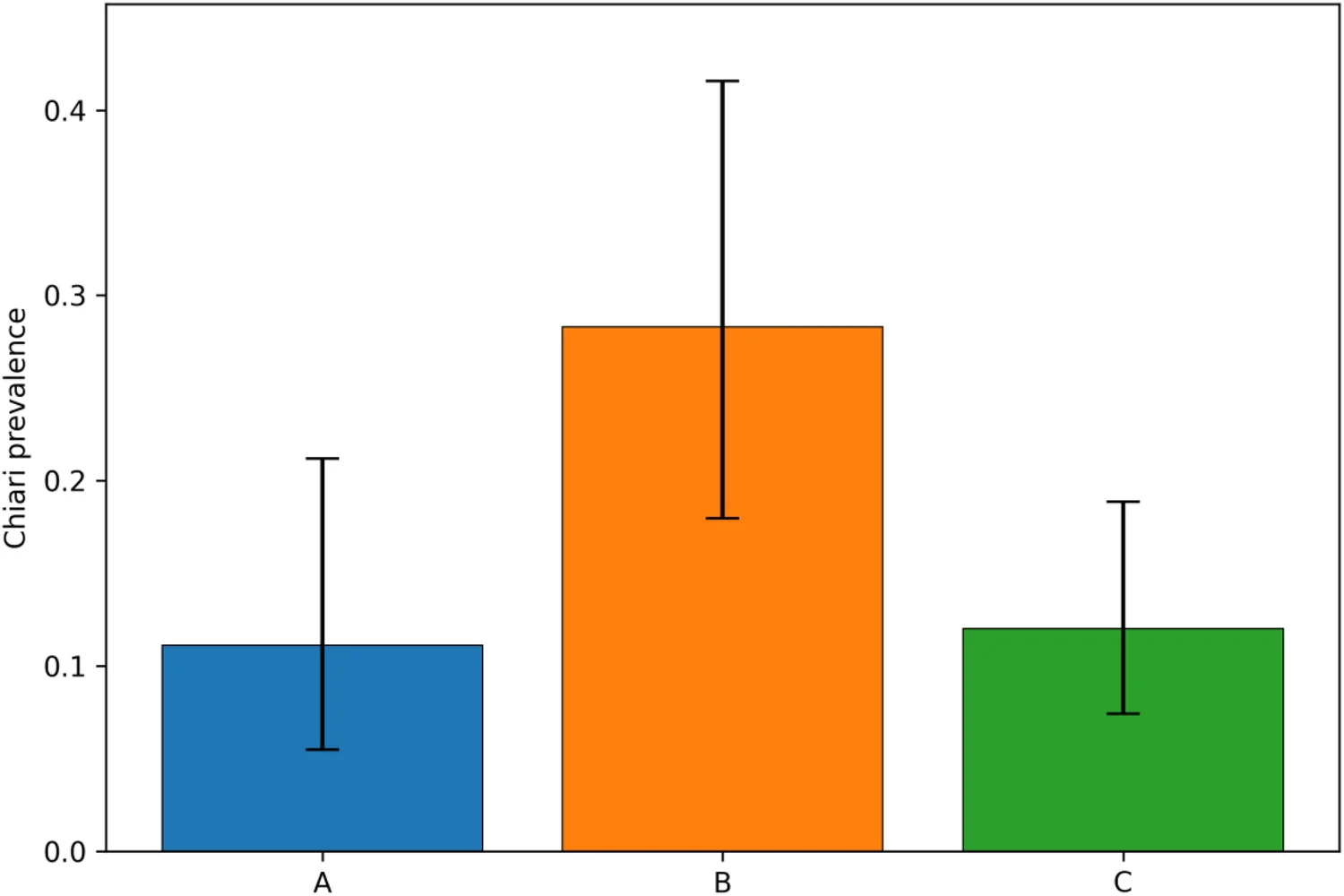
Prevalence of chiari type I malformation across sagittal synostosis subtypes. Legend: Bar plot comparing preoperative CM-I prevalence in subtype A (blue), B (orange), and C (green). Error bars indicate 95% confidence intervals. Subtype A: sagittal synostosis with anterior fossa involvement; subtype B: sagittal synostosis with posterior fossa involvement; subtype C: isolated sagittal synostosis.

To characterize the overall association between anatomical subtype and CM-I without conditioning on posterior fossa morphometric variables, a subtype-only logistic regression model was first constructed (Model 1). Using Group C as the reference, Group B was associated with significantly higher odds of CM-I (OR = 2.89, 95% CI 1.29–6.48; *P* = 0.010), whereas no significant difference was observed between Groups A and C (OR = 0.92, 95% CI 0.35–2.38; *P* = 0.858). These findings demonstrate a greater overall CM-I burden in the posterior fossa–involved phenotype before adjustment for quantitative posterior fossa morphology (Table 2).

**Table 2:** Sequential logistic regression analyses of anatomical subtype and posterior fossa morphologic factors associated with chiari type I malformation.

Model	Variable	OR (95% CI)	P
Model 1: subtype-only	A vs. C	0.92 (0.35–2.38)	0.858
	B vs. C	2.89 (1.29–6.48)	0.010
	AUC	0.614	—
Model 2: original base multivariable model	A vs. C	0.29 (0.11–0.73)	0.009
	B vs. C	0.29 (0.10–0.86)	0.025
	Tentorial angle, per 5°	3.10 (2.17–4.42)	<0.001
	Evans index, per 0.05	1.15 (0.38–3.46)	0.807
	Age at surgery, per 12 mo	1.03 (0.73–1.46)	0.858
	Hydrocephalus	3.52 (0.25–49.71)	0.351
	AUC	0.869	—
Model 3: Model 2 + PFV_adj	A vs. C	0.28 (0.10–0.72)	0.009
	B vs. C	0.20 (0.06–0.64)	0.007
	Tentorial angle, per 5°	3.20 (2.10–4.88)	<0.001
	Evans index, per 0.05	1.52 (0.45–5.07)	0.497
	Age at surgery, per 12 mo	0.97 (0.68–1.40)	0.884
	Hydrocephalus	2.42 (0.15–39.96)	0.537
	PFV_adj, per 10 mL	0.74 (0.62–0.89)	0.002
	AUC	0.885	—

ORs and 95% CIs are shown. Model 1 included subtype only; Model 2 included subtype, tentorial angle, Evans index, age at surgery, and hydrocephalus; Model 3 additionally included PFV_adj. Subtype C was the reference. Continuous predictors were scaled per 5° (tentorial angle), 0.05 (Evans index), 12 months (age), and 10 mL (PFV_adj). PFV_adj was defined as the residual from PFV∼ICV_pre + age. Subtype coefficients in Models 2–3 represent morphology-conditioned associations rather than overall subtype effects. OR, odds ratio; CI, confidence interval; AUC, area under the ROC curve; PFV, posterior fossa volume; ICV_pre, preoperative intracranial volume.

### 3.3 Association between PFV and ICV and covariate-adjusted assessment

Scatterplots and ANCOVA modeling demonstrated a positive association between PFV and preoperative ICV (ICV) in all subtypes, with PFV increasing as ICV increased. After fixing age at surgery at the cohort mean, the subtype-specific fitted curves showed clear spatial separation (Figure 2), indicating systematic differences in cranial volumetric allocation across phenotypes. Consistently, covariate-adjusted PFV estimated by least-squares means (LS-means) was significantly lower in Group B than in Groups A and C under comparable physiological conditions (Figure 3). In line with this, the residual distribution of PFV_adj revealed a markedly negative shift in Group B, with the median well below zero, isolating a structural signal of relative posterior fossa deficiency after accounting for developmental background (Figure 4).

**Figure 2:**
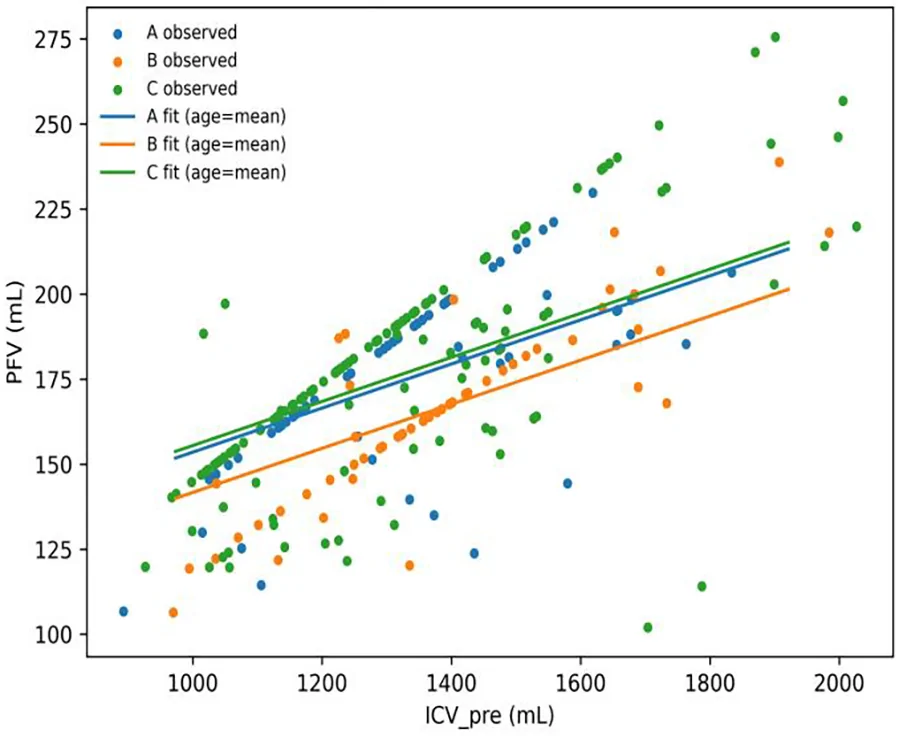
Association between posterior fossa volume (PFV) and preoperative intracranial volume (ICV). Legend: Each point represents an individual. The PFV–ICV relationship was modeled using ANCOVA with adjustment for age at surgery; fitted lines show subtype-specific predictions with age fixed at the cohort mean. Colors: subtype A (blue), B (orange), C (green).

**Figure 3:**
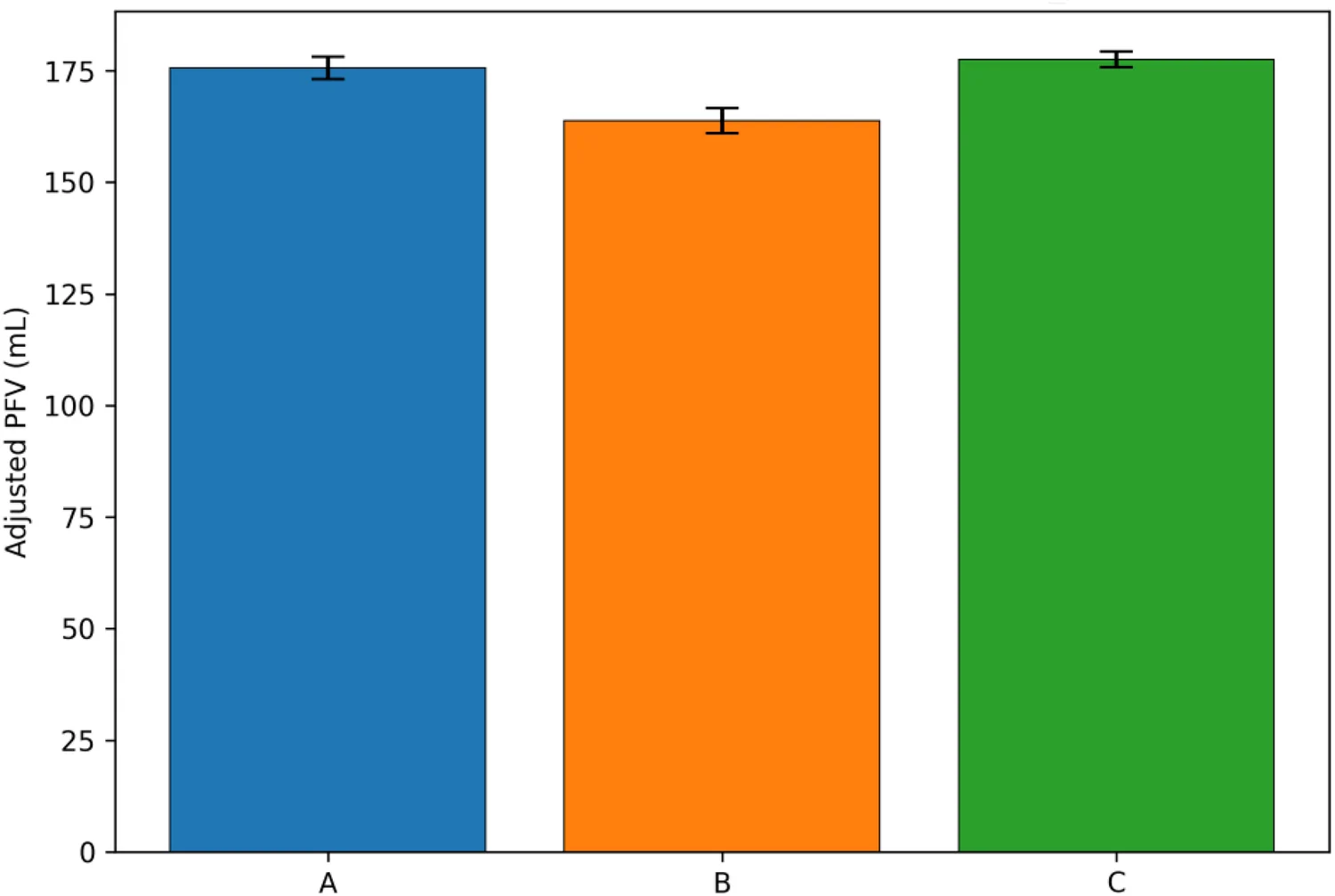
Covariate-adjusted PFV differences across sagittal synostosis subtypes (LS-means). Legend: PFV was modeled by ANCOVA (PFV as outcome; ICV and age at surgery as covariates; subtype as the main factor). Bars represent least-squares means (LS-means) estimated at the cohort-mean ICV and age; error bars indicate standard error (SE). Colors: A (blue), B (orange), C (green).

**Figure 4:**
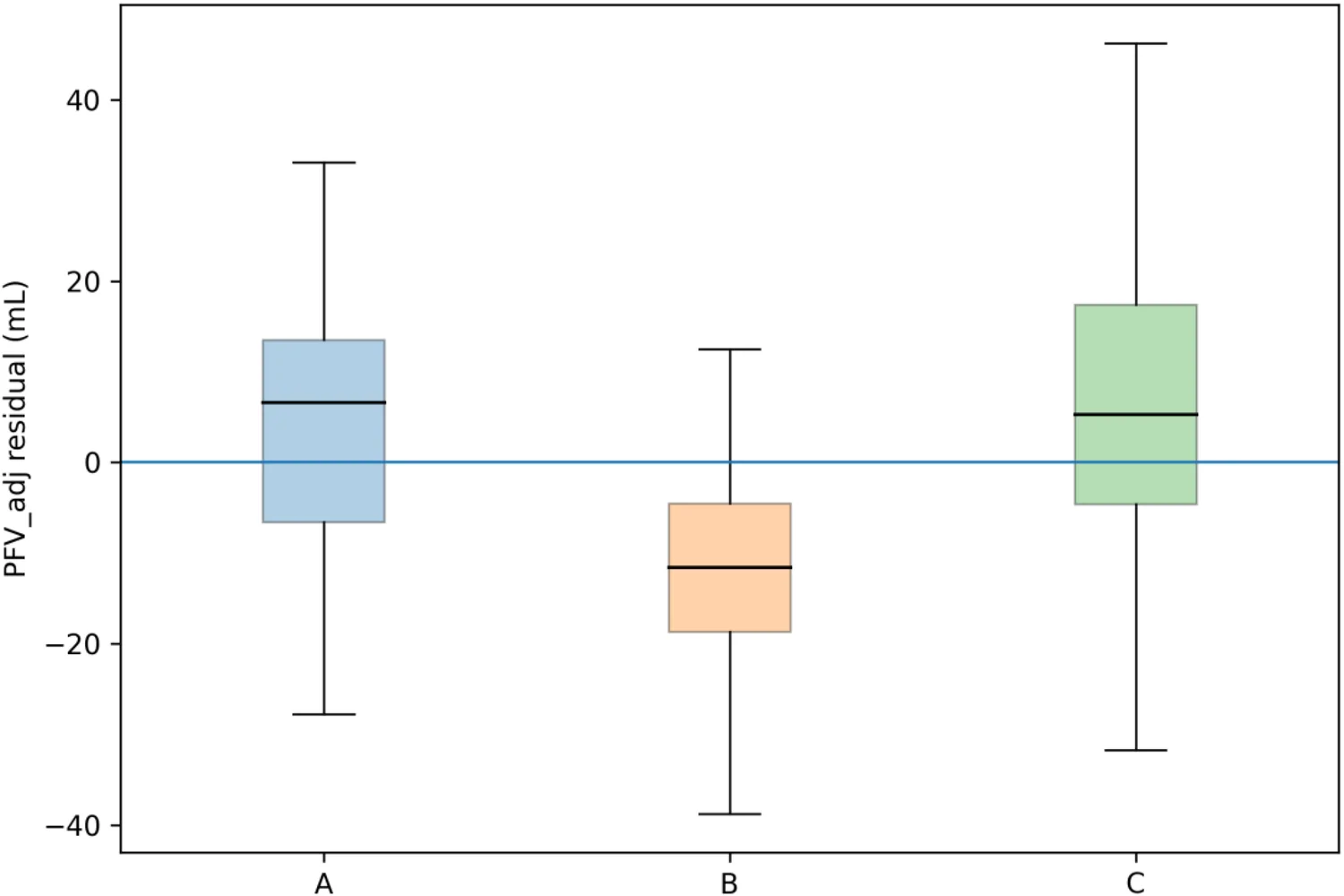
Distribution of residualized posterior fossa volume (PFV_adj) across subtypes. Legend: Boxplots show PFV_adj, defined as the residual from a linear regression model with PFV as the dependent variable and ICV_pre and age at surgery as covariates (mL), representing deviation from expected PFV after accounting for cranial size and age. Values <0 indicate relative posterior fossa deficiency. Boxes denote P25–P75 with the median line; whiskers represent 1.5 ×  IQR. The horizontal line marks 0. Colors: A (blue), B (orange), C (green).

### 3.4 Morphology-adjusted associations with CM-I

We next examined CM-I associations after conditioning on quantitative posterior fossa morphology. Model 2 included anatomical subtype, tentorial angle, Evans index, age at surgery, and hydrocephalus. Tentorial angle showed the strongest and most consistent association with CM-I. Each 5° increase in tentorial angle was associated with approximately threefold higher odds of CM-I (OR = 3.10, 95% CI 2.17–4.42; *P* < 0.001). Evans index (OR = 1.15 per 0.05 increase, 95% CI 0.38–3.46; *P* = 0.807), age at surgery (OR = 1.03 per 12-month increase, 95% CI 0.73–1.46; *P* = 0.858), and hydrocephalus (OR = 3.52, 95% CI 0.25–49.71; *P* = 0.351) were not significantly associated with CM-I.

In Model 3, which additionally incorporated PFV_adj, the association between tentorial angle and CM-I remained robust (OR = 3.20 per 5° increase, 95% CI 2.10–4.88; *P* < 0.001). PFV_adj was inversely associated with CM-I, with each 10-mL increase corresponding to lower odds of CM-I (OR = 0.74, 95% CI 0.62–0.89; *P* = 0.002). Evans index (OR = 1.52, 95% CI 0.45–5.07; *P* = 0.497), age at surgery (OR = 0.97, 95% CI 0.68–1.40; *P* = 0.884), and hydrocephalus (OR = 2.42, 95% CI 0.15–39.96; *P* = 0.537) remained nonsignificant (Table 2; Figure 6).

Notably, the association of Group B with CM-I changed substantially across the sequential models. Compared with Group C, Group B was associated with higher CM-I odds in the subtype-only Model 1 (OR = 2.89, 95% CI 1.29–6.48; *P* = 0.010), whereas the conditional OR decreased below unity after adjustment for tentorial angle and other morphologic characteristics in Model 2 (OR = 0.29, 95% CI 0.10–0.86; *P* = 0.025) and Model 3 (OR = 0.20, 95% CI 0.06–0.64; *P* = 0.007). Given that Group B represents a posterior fossa–involved phenotype and that tentorial angle and PFV_adj quantitatively capture major structural components of this phenotype, this reversal was not interpreted as evidence of a protective effect of Group B. Rather, it is consistent with conditioning on morphologic features that may account for a substantial component of the overall subtype-associated CM-I difference.

### 3.5 Model discrimination

Model 2 demonstrated good discrimination for CM-I, with an AUC of 0.869. After PFV_adj was added, the AUC increased numerically to 0.885 in Model 3 (Figure 5). Thus, incorporation of PFV_adj was associated with a modest numerical improvement in model discrimination. Given the absence of external validation and a prespecified statistical comparison of AUCs, this difference was not interpreted as evidence of a statistically established incremental predictive benefit.

**Figure 5:**
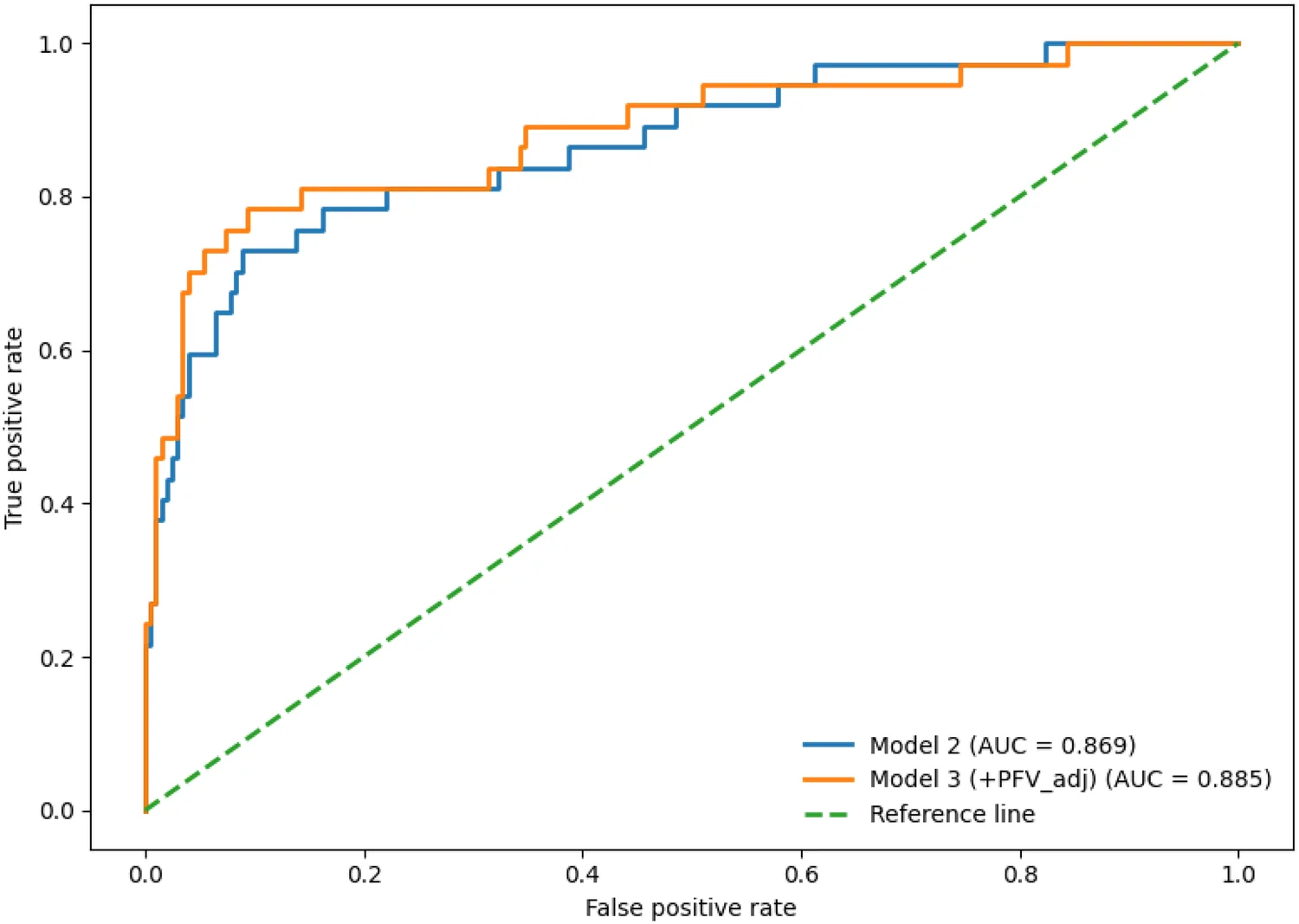
Receiver operating characteristic curves comparing CM-I discrimination between model 2 and model 3. Legend: ROC curves comparing the discrimination of Model 2 and Model 3 for CM-I. Model 3 additionally included PFV_adj. AUCs were 0.869 and 0.885, respectively. The dashed diagonal line indicates chance-level discrimination.

**Figure 6:**
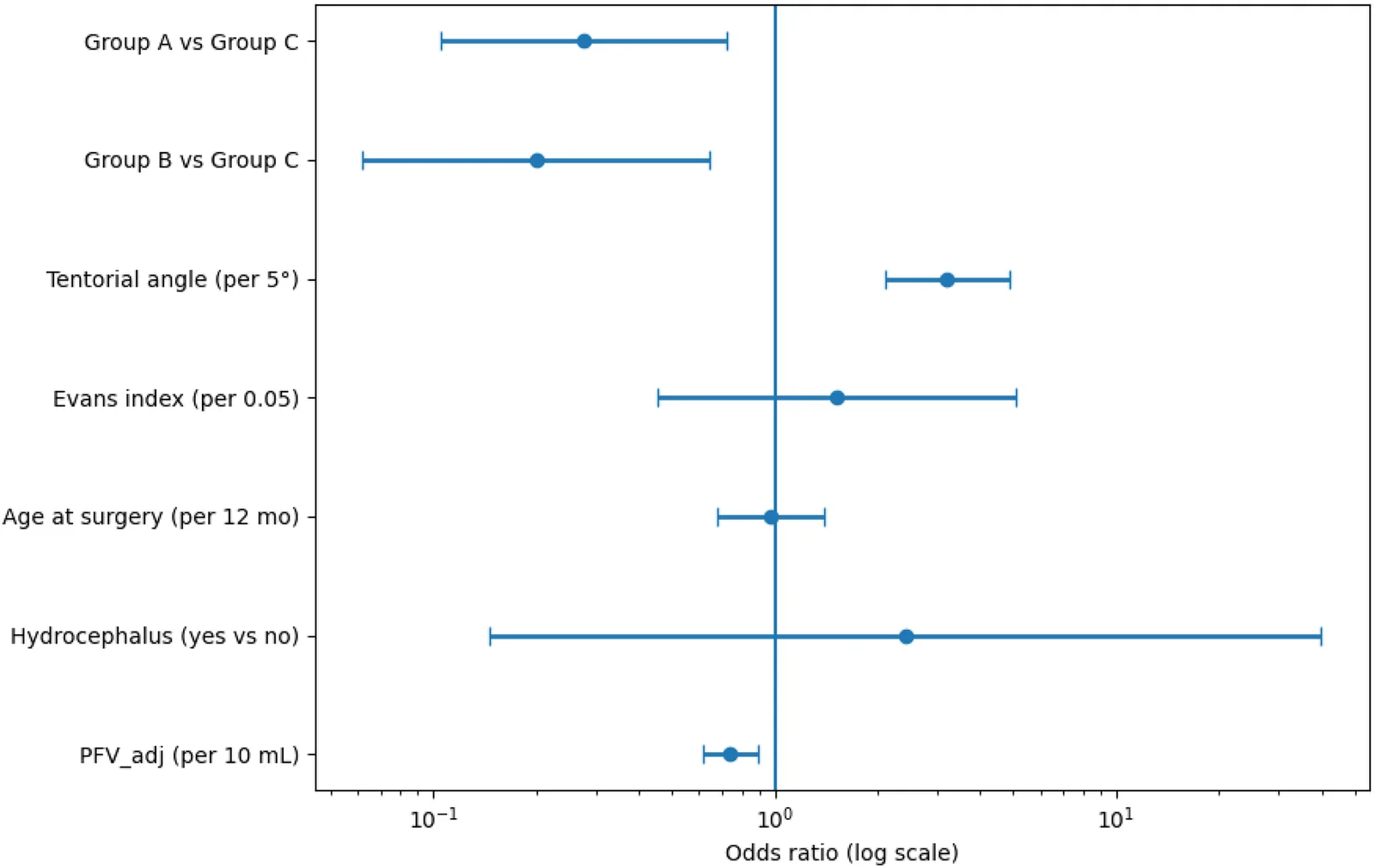
Conditional multivariable associations with chiari type I malformation in model 3. Legend: Forest plot showing odds ratios (ORs) and 95% confidence intervals (CIs) from Model 3. Subtype C was the reference group. Continuous predictors were scaled per 5° for tentorial angle, 0.05 for Evans index, 12 months for age at surgery, and 10 mL for PFV_adj. The vertical line indicates OR = 1.

### 3.6 Sensitivity analysis using firth penalized logistic regression

A total of 37 CM-I events were observed. The events-per-non-intercept-parameter ratios were 18.5 for Model 1, 6.2 for Model 2, and 5.3 for Model 3. Given the relatively low EPV in the multivariable models, Firth bias-reduced penalized logistic regression was performed as a sensitivity analysis.

The overall subtype association was preserved in Firth Model 1: Group B remained associated with higher odds of CM-I compared with Group C (OR = 2.87, 95% CI 1.29–6.38; *P* = 0.010), whereas Group A did not differ significantly from Group C (OR = 0.95, 95% CI 0.37–2.41; *P* = 0.908).

The principal morphologic associations were also preserved in the penalized models. In Firth Model 3, each 5° increase in tentorial angle was associated with higher CM-I odds (OR = 2.93, 95% CI 1.97–4.35; *P* < 0.001), whereas each 10-mL increase in PFV_adj was associated with lower CM-I odds (OR = 0.76, 95% CI 0.63–0.92; *P* = 0.006). The estimate for hydrocephalus remained imprecise (OR = 2.25, 95% CI 0.26–19.50; *P* = 0.462), consistent with the small number of hydrocephalus events. Full results of the Firth sensitivity analysis are provided in Supplementary Table S1.

## 4 Disscusion

Using imaging-derived measurements and clinical outcomes from 241 children with sagittal synostosis, this study delineates a structural link between anatomical subtypes of sagittal synostosis and the risk of Chiari type I malformation (CM-I). The primary contribution is not a refined point estimate of overall CM-I prevalence, but evidence of meaningful within-cohort heterogeneity: posterior fossa–involved morphology identifies a distinctly enriched high-risk subgroup, with CM-I prevalence exceeding that typically reported for isolated sagittal synostosis (ISS). These results provide a quantitative basis for preoperative risk stratification and tailored imaging surveillance, consistent with emerging views that sagittal synostosis represents a phenotypic spectrum with divergent compensatory patterns rather than a single entity (15).

Prior literature generally indicates that craniosynostosis-associated CM-I is more prevalent in multisuture or syndromic craniosynostosis and in certain single-suture phenotypes—particularly lambdoid synostosis—whereas isolated sagittal synostosis tends to show a lower, yet non-negligible, prevalence. Strahle et al. reported that, in a single-center cohort of 383 patients with craniosynostosis, the prevalence of CM-I among those with sagittal synostosis was approximately 3%, while higher rates were observed in lambdoid and pansynostosis phenotypes; they further noted that CM-I and syringomyelia may improve after craniofacial correction in some patients, whereas *de novo* postoperative CM-I can also occur, supporting a dynamic component to this entity (8). In a separate cohort of 377 patients with isolated sagittal synostosis (ISS), Davis et al. reported an overall CM-I prevalence of 5.8% and demonstrated a markedly higher CM-I proportion among those presenting later or at older ages, underscoring the possibility of progression over time and supporting adjunct screening for raised intracranial pressure in ISS (9). The CM-I prevalence observed in our cohort (15.4%) exceeded that typically reported for ISS, likely reflecting enrichment of posterior fossa–involved phenotypes, routine MRI-based detection, and cohort heterogeneity. Rather than merely revising prevalence estimates, our data identify a morphologically distinct subgroup with a substantially greater CM-I burden, supporting a more granular, phenotype-driven approach to preoperative evaluation in sagittal synostosis.

The classical pathobiological framework posits that the central mechanism of Chiari type I malformation (CM-I) is an absolute or relative insufficiency of posterior fossa volume and/or compensatory compliance (8, 9), which subsequently promotes tonsillar herniation and abnormal cerebrospinal fluid (CSF) dynamics at the foramen magnum (16, 17). In parallel, the concept of “acquired Chiari” has been proposed, whereby secondary tonsillar descent occurs under conditions such as constrained cranial growth, CSF diversion, or aberrant intracranial pressure gradients (18–20). In our cohort, Group B demonstrated a systematic negative shift in PFV_adj, and higher PFV_adj was associated with lower odds of CM-I in the morphology-adjusted model. These findings support relative posterior fossa deficiency as an important anatomical feature associated with CM-I. Importantly, CM-I occurrence therefore appears not to be determined solely by the absolute physical size of PFV or ICV; rather, after accounting for individual developmental context, including age and overall cranial volume, relative posterior fossa underdevelopment provides additional anatomical information.

An important issue raised by the sequential models concerns the interpretation of the subtype coefficients. In the subtype-only Model 1, Group B was associated with significantly higher CM-I odds than Group C (OR = 2.89), consistent with its higher crude CM-I prevalence. However, the Group B coefficient fell below unity after adjustment for tentorial angle and other morphologic characteristics in Model 2 and remained below unity after PFV_adj was added in Model 3. This reversal should not be interpreted as evidence that Group B becomes protective after adjustment. Rather, Group B is itself defined by posterior cranial involvement, while tentorial configuration and relative posterior fossa volume quantitatively capture structural features closely related to this phenotype. Conditioning on these variables therefore removes part of the morphological pathway through which the Group B phenotype may be associated with CM-I and can substantially attenuate or even reverse the subtype coefficient. Accordingly, tentorial angle and PFV_adj are more appropriately interpreted as quantitative morphologic pathway variables rather than simply independent add-on predictors. Although this pattern is compatible with a potential mediating anatomical pathway, the present retrospective observational study was not designed to establish formal causal mediation.

The strong association of tentorial angle provides an additional, anatomy-driven line of evidence. Multiple morphometric studies have reported a steeper tentorial configuration and/or an altered cranial base angular profile in CM-I, consistent with a developmental shift in the osseous cranial base–posterior fossa framework (8, 21). Karagöz et al., for example, described a steeper tentorial angle relative to the Twining line in CM-I patients (22). Sekula et al. further observed, in individuals with Chiari-like symptoms but subthreshold tonsillar descent, that increased tentorial angle co-occurred with underdevelopment of posterior fossa bony structures, reinforcing the notion that the “posterior fossa osseous framework + tentorial configuration” jointly contributes to foramen magnum crowding (23). Accordingly, our results support tentorial angle remained strongly associated with CM-I risk—potentially reflecting an upstream, relatively stable anatomical trait of posterior fossa architecture—whereas adjusted posterior fossa volume provides a complementary volumetric quantification of this architecture. Together, these metrics offer a coherent anatomical explanation for posterior fossa crowding. Notably, the direction and statistical significance of tentorial angle differences have been heterogeneous across studies, and systematic reviews/meta-analyses suggest that certain morphometric parameters (including tentorium-related angles) are not uniformly significant in all cohorts, likely reflecting differences in population structure, measurement definitions, and confounder-adjustment strategies (24).

A key contribution of this study is demonstrating that, even when ICV is comparable across subtypes, unadjusted PFV may show only modest between-group differences, whereas PFV_adj—after adjustment for ICV and age—substantially enhances subtype separation. This approach is biologically motivated by the dynamic nature of posterior fossa development: during infancy and early childhood, posterior fossa growth is strongly age-dependent and follows a non-linear trajectory (23, 25, 26). This approach is biologically motivated by the dynamic nature of posterior fossa development: during infancy and early childhood, posterior fossa growth is strongly age-dependent and follows a non-linear trajectory (27, 28). Prior work has often relied on the PFV/ICV ratio to mitigate confounding by body size; however, ratio-based normalization has important limitations (29). In particular, simple ratios do not adequately address age-related bias under non-linear growth, nor do they capture the complex covariance structure among age, ICV, and PFV. In contrast, the residual method used here fits a regression model with ICV and age as predictors, thereby quantifying the extent to which an individual's PFV deviates from its expected developmental value under a common physiological reference (30). This strategy is statistically more robust and more directly aligned with the clinical construct of “relative underdevelopment,” providing a developmentally adjusted measure of relative posterior fossa underdevelopment.

The clinical implications of our findings primarily relate to earlier screening and refined risk stratification. First, patients with the posterior fossa–involved phenotype (Type B), low PFV_adj, and/or a steep tentorial angle may warrant more systematic imaging evaluation for CM-I, including MRI when indicated and targeted assessment for associated complications, with particular attention to postoperative evolution. Prior studies suggest that CM-I may progress with age and is more common among later-presenting subgroups, and have advocated adjunct fundoscopic examination to screen for signs related to elevated intracranial pressure (ICP) (9, 31). Second, the instability of the Evans index in our models and the wide confidence intervals for hydrocephalus indicate that ventricular morphology alone is insufficient to replace posterior fossa phenotyping for CM-I risk explanation. This does not, however, diminish the potential relevance of ICP. Existing evidence suggests that intracranial hypertension in craniosynostosis may synergize with CM-I risk within the same anatomical compartment, and papilledema represents a key clinical marker of raised ICP (32). In addition, delayed postoperative intracranial hypertension merits vigilance; systematic reviews have indicated that late-onset intracranial hypertension after craniosynostosis surgery is not uncommon and may require long-term follow-up and, when appropriate, monitoring (33). Larger, multicenter prospective studies are needed to further validate and clarify the pathophysiological cascade from ventricular dilatation to abnormal ICP gradients and subsequent tonsillar descent.

Several limitations should be acknowledged. First, although volumetric and angular measurements enabled quantitative assessment of posterior fossa morphology and cranial base geometry, measurement variability may arise from differences in imaging modality, segmentation procedures, and interpretive thresholds. Second, the lower gestational age observed in Group B suggests that perinatal developmental context may influence posterior fossa growth trajectories; future studies should incorporate gestational age and prematurity-related variables more comprehensively. Third, the lack of longitudinal postoperative imaging precluded direct evaluation of dynamic outcomes, including CM-I resolution after cranial remodeling or *de novo* postoperative CM-I.

Fourth, the number of CM-I events was modest relative to the number of parameters included in the multivariable models, with events-per-parameter ratios of approximately 6.2 and 5.3 for Models 2 and 3, respectively. This may reduce the precision of conventional maximum-likelihood estimates, particularly for infrequent predictors such as hydrocephalus. We therefore performed Firth penalized logistic regression as a sensitivity analysis. The principal associations involving tentorial angle and PFV_adj remained consistent, supporting the robustness of the main morphologic findings, although individual subtype coefficients in the fully adjusted models should still be interpreted cautiously.

Fifth, because subtype assignment was retrospectively derived from imaging and operative records, formal blinding to CM-I status could not be guaranteed in all cases. Although CM-I status itself and quantitative PFV_adj or tentorial-angle thresholds were not used as subtype classification criteria, residual classification or incorporation bias cannot be completely excluded. Finally, the attenuation and reversal of subtype coefficients after morphologic adjustment should not be regarded as proof of causal mediation. Future multicenter prospective cohorts with standardized MRI acquisition, harmonized volumetric and angular measurement protocols, longitudinal follow-up, and prespecified mediation analyses will be required to validate these findings and further clarify the anatomical pathway linking sagittal synostosis phenotype to CM-I.

In conclusion, sagittal synostosis comprises anatomically distinct phenotypes with heterogeneous CM-I burden. The posterior fossa–involved phenotype showed the highest overall CM-I burden and was characterized by relative posterior fossa volume deficiency and a steeper tentorial configuration. The marked attenuation and reversal of subtype coefficients after morphologic adjustment suggest that these quantitative posterior fossa characteristics may account for an important component of the subtype-associated CM-I difference, rather than indicating a protective effect of the posterior fossa–involved phenotype after adjustment. Residualized PFV provides a developmentally adjusted measure of relative posterior fossa underdevelopment and may complement conventional anatomical subtype classification.

## Data Availability

The original contributions presented in the study are included in the article/Supplementary Material, further inquiries can be directed to the corresponding authors.
